# The structural biology of canonical Wnt signalling

**DOI:** 10.1042/BST20200243

**Published:** 2020-07-29

**Authors:** Mark Agostino, Sebastian Öther-Gee Pohl

**Affiliations:** 1 School of Pharmacy and Biomedical Sciences, Curtin Health Innovation Research Institute (CHIRI), Curtin University, Bentley, Western Australia, Australia; 2 Curtin Institute for Computation, Curtin University, Bentley, Western Australia, Australia; 3 The Institute of Genetics and Molecular Medicine, Edinburgh Cancer Research Centre, University of Edinburgh, U.K.

**Keywords:** beta-catenin, cancer, crystallography, frizzled, glycogen synthase kinase, Wnt proteins

## Abstract

The Wnt signalling pathways are of great importance in embryonic development and oncogenesis. Canonical and non-canonical Wnt signalling pathways are known, with the canonical (or β-catenin dependent) pathway being perhaps the best studied of these. While structural knowledge of proteins and interactions involved in canonical Wnt signalling has accumulated over the past 20 years, the pace of discovery has increased in recent years, with the structures of several key proteins and assemblies in the pathway being released. In this review, we provide a brief overview of canonical Wnt signalling, followed by a comprehensive overview of currently available X-ray, NMR and cryoEM data elaborating the structures of proteins and interactions involved in canonical Wnt signalling. While the volume of structures available is considerable, numerous gaps in knowledge remain, particularly a comprehensive understanding of the assembly of large multiprotein complexes mediating key aspects of pathway, as well as understanding the structure and activation of membrane receptors in the pathway. Nonetheless, the presently available data affords considerable opportunities for structure-based drug design efforts targeting canonical Wnt signalling.

## Brief overview of canonical Wnt signalling

**Figure 1.**
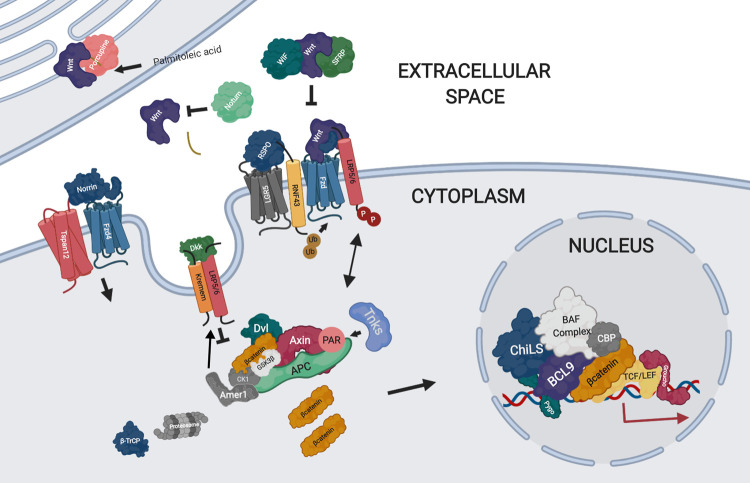
Graphical overview of canonical Wnt signalling. Figure prepared using BioRender.

Wnt signalling involves a series of complex pathways and underpins developmental processes [1]. When dysregulated, it is synonymous with impaired regeneration and a variety of pathological states, including carcinogenesis [2–5]. Wnt signalling is primarily classed into canonical (β-catenin dependent) and non-canonical (β-catenin independent) pathways. Canonical Wnt signalling is primarily controlled through the regulation of three distinct multiprotein complexes: the signalosome, the degradosome and the nuclear enhanceosome, as well a variety of extracellular agonists and antagonists which precede these intracellular events [6] (Figure 1).

Wnt signalling can be initiated or enhanced by a variety of extracellular ligands, including Wnt and Norrin proteins, which bind to Frizzled (Fzd) receptors, and R-spondins (RSPOs), which bind to LGR family receptors. Wnt proteins are lipid-modified at a conserved serine by the *O*-acyltransferase Porcupine to facilitate secretion and receptor binding. Canonical Wnt signalling can be amplified following concomitant binding of Wnt and R-spondin (RSPO) ligands, which may function dependently or independently of LGR [7]. RSPOs prevents Fzd degradation by blocking the activity of the RING finger ubiquitin ligases, RNF43 and ZNRF3 [8,9]. Norrin is an atypical Wnt ligand that can bind specifically to Fzd4 and LRP5/6 [10], as well as the Fzd4–Tspan12 complex, to activate Wnt signalling [11]. Extracellular antagonists include Wnt inhibitory factor (WIF), secreted-Frizzled related proteins (sFRPs), Dickkopfs (DKKs) and Notum, each of which are diverse in structure and function (specific details of which will be elaborated later in the review). Wnt ligand binding to membrane-bound receptors and co-receptors results in the formation of multiprotein assemblies or ‘signalosomes’, composed of Fzd receptors and LRP5/6 co-receptors bound to Wnt ligands. These signalosomes are highly dynamic and can be negatively regulated by RNF43/ZNRF3, which, in turn, is balanced by R-spondin–LGR5 receptor interactions [12].

Intracellularly, Wnt signalling is controlled at the level of the β-catenin destruction complex, or the degradosome, which primarily consists of the scaffold protein Axin, glycogen synthase kinase 3β (GSK3β), casein kinase 1α (CK1α), protein phosphatase 2A (PP2A) and Adenomatous Polyposis Coli (APC). [13] In the absence of Wnt stimulation, β-catenin is sequentially phosphorylated by CK1α (Ser45) and GSK3β (Thr41, Ser37, Ser33), resulting in ubiquitin-mediated proteasomal degradation through a β-TrCP-dependent mechanism. Following Wnt stimulation, the degradosome is recruited to the membrane through a Dvl-Axin mediated mechanism, where phosphorylation of the co-receptor LRP5/6 on its cytoplasmic tail by GSK3β and CK1α/ε occurs [13]. The recruitment of GSK3β/CK1 and Axin can be mediated by adenomatous polyposis coli membrane recruitment 1 (Amer1) [14]. This, in turn, can result in the inhibition of GSK3β [15–18] and the translocation of β-catenin to the nucleus. Poly(ADP-ribosyl)ation of Axin by Tankyrase mediates its ubiquitination and subsequent degradation, destabilising the destruction complex, and thus activating Wnt signalling [19]. Once localised to the nucleus, β-catenin acts a co-factor for the initiation of the transcription of Wnt target genes [2]. This Wnt-driven transcriptional program is controlled by the Wnt enhanceosome, the core of which is made up of the ChiLS (Chip-SSDP/LIM-domain binding protein) complex, which binds Pygopus, Groucho/TLE and scaffold protein BCL9/legless [20,21]. In a ‘Wnt off’ context Groucho/TLE binds TCF/LEF and ChiLS to repress transcription, while in a ‘Wnt on’ environment, β-catenin induces an enhanceosome complex rearrangement to bind to TCF/LEF transcription factors, and other transcriptional co-activators (e.g. CREB-binding protein and BAF complex) to initiate target gene expression [22].

## Structural knowledge of extracellular regulation of Wnt signalling

### Wnts and related proteins

**Figure 2.**
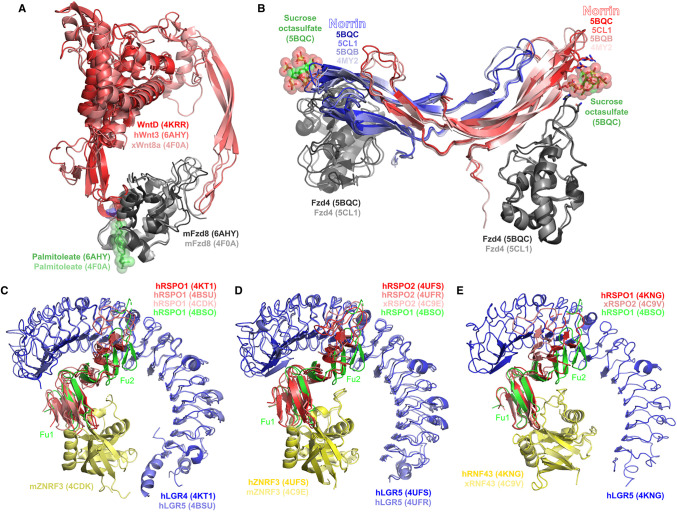
Structures related to interactions involving secreted positive regulators of Wnt signalling. (**A**) Wnt-related proteins and interactions. Structures depicted include: WntD (PDB 4KRR); the human Wnt3 complex with the mouse Fzd8 cysteine-rich domain (PDB 6AHY); the *Xenopus* Wnt8a complex with the mouse Fzd8 cysteine-rich domain (PDB 4F0A). (**B**) Norrin and its interactions. Structures depicted include: unbound norrin-maltose binding protein fusion (PDB 4MY2), unbound norrin (PDB 5BQB), norrin-maltose binding protein fusion in complex with Fzd4 (PDB 5CL1), norrin-Fzd4-sucrose octasulfate ternary complex (PDB 5BQC). Maltose binding protein hidden. Key residues contacting sucrose octasulfate in PDB 5BQC are shown as sticks. (**C**) RSPO1–LGR–ZNRF3 complexes. Structure represented include: human RSPO1–LGR4 complex (PDB 4KT1), human RSPO1–LGR5 complex (PDB 4BSU), human RSPO1 in complex with mouse ZNRF3 (PDB 4CDK). The structure of native human RSPO1 (PDB 4BSO) is shown for reference. (**D**) RSPO2–LGR–ZNRF3 complexes. Structures represented include: human RSPO2–LGR5–ZNRF3 complex (PDB 4UFS), human RSPO2–LGR5 complex (PDB 4UFR), *Xenopus* RSPO2 in complex with mouse ZNRF3 (PDB 4C9E). The structure of native human RSPO1 (PDB 4BSO) is shown for reference. (**E**) R-spondin–LGR–RNF43 complexes. Structures represented include: human LGR5–RSPO1–RNF43 complex (PDB 4KNG); *Xenopus* RSPO2–RNF43 complex (PDB 4C9V). The native human RSPO1 (PDB 4BSO) is shown for reference.

The structures of a relatively limited number of Wnt protein family members have been solved (Figure 2A). This is due in part to the presence of *O*-lipidation at a conserved serine that makes Wnt proteins highly hydrophobic and challenging to purify. The first structure of a Wnt protein solved was the *Xenopus* Wnt8 in complex with the mouse Fzd8 cysteine-rich domain (CRD), revealing a novel protein fold and the importance of lipidation for direct binding of Fzd [23]. This structure further illustrated that Wnts bind to Fzds at two distinct sites on opposite faces of the CRD. The Wnt protein family contains 19 members in mammals, however, the structure of only one mammalian Wnt has been solved experimentally [24]; further study of the Wnt family in mammals has been facilitated by computational approaches [25–27]. The structure of the N-terminal region of the *Drosophila* WntD protein revealed an overall similar fold to the N-terminal regions of other members of the Wnt family [28], although unlike other members of the family, this protein is not lipidated [29].

### Norrin

Norrin is an atypical Wnt signalling activator displaying a distinct fold to Wnt, achieving its activity through forming a ternary complex with a Fzd and an LGR (Figure 2B). The first structure of Norrin obtained was that of its fusion with maltose binding protein [10]; a complex of this fusion protein with the Fzd4 CRD was subsequently obtained [30]. The structures of the unfused Norrin, its complex with Fzd4, and the Norrin-Fzd4 ternary complex with the heparin mimic sucrose octasulfate have also been determined [31], revealing the potential for glycosaminoglycans to bridge the Norrin-Fzd4 interaction. Specifically, norrin residues Lys58, Arg107, Arg109 and Arg115 and Fzd4 residues His154 and Asn155 interact directly with sucrose octasulfate in the crystal structure (Figure 2B).

### R-spondins (RSPOs)

RSPOs feature two furin repeat domains (Fu1 and Fu2), as illuminated in the structures of the human RSPO1 and the *Xenopus* RSPO2 [32,33], and between which considerable flexibility is observed (Figure 2C–E). Numerous structures of RSPO1 bound to the leucine-rich repeat (LRR) ectodomains of LGR4 and LGR5 have been reported [33–35]; a structure of RSPO2 bound to the LGR5 ectodomain has also been reported [36]. In all cases, these structures feature the LRRs of the LGR ectodomain curving around Fu2 of the RSPO. Fu1 of RSPOs mediates the interaction with ZNRF3 [32–37] and RNF43 [32]. Due to the complementary utilisation of the Fu1 and Fu2 domains, ternary complexes of RSPOs, LGRs and RING finger ubiquitin ligases are possible and have been structurally characterised [36–38]. Subtle variations in how ZNRF3 and RNF43 are recognised by LGRs are observed in the crystal structures; specifically, the LRR does not appear to directly bind ZNRF3 (although in one structure, a helix immediately after the LRR is observed to bind to ZNRF3) (Figure 2C,D), while RNF43 is directly bound by the LRR, albeit weakly (Figure 2E).

### Secreted Frizzled-related proteins (sFRPs)

**Figure 3.**
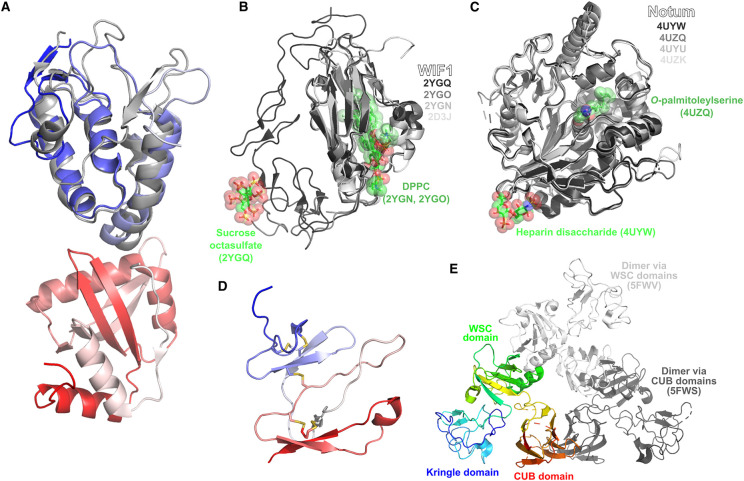
Structures related to interactions involving secreted negative regulators of Wnt signalling. (**A**) Secreted Frizzled-related proteins. Legend: grey — mouse sFRP3 cysteine-rich domain (PDB 1IJX); blue-white-red N-to-C-terminal — *Xenopus* Sizzled (PDB 5XGP). (**B**) Wnt Inhibitory Factors (WIFs). Structures depicted include: NMR structure of WIF domain of native human WIF1 (PDB 2D3J); WIF domain of human WIF1 bound to dipalmitoylphosphatidylcholine (DPPC) (PDB 2YGN); WIF domain and epidermal growth factor-like (EGF-like) domain 1 of human WIF1 (PDB 2YGO); WIF domain and EGF-like domains 1–3 of human WIF bound to DPPC and sucrose octasulfate (PDB 2YGQ). (**C**) Notum. Structures depicted include: native *Drosophila* Notum (PDB 4UZK); native human Notum (PDB 4UYU); human Notum bound to *O*-palmitoleylserine (PDB 4UZQ); human Notum bound to heparin disaccharide (ΔUA(2S)α1-4GlcNS(6S) (PDB 4UYW). (**D**) NMR structure of mouse Dickkopf-2 (PDB 2JTK). Structure coloured from N-to-C-terminal by blue-white-red gradient. (**E**) Dimer formation by Kremen1. Monomeric Kremen1 (PDB 5FWU) is depicted as red-to-blue N-to-C terminal rainbow, with positions of second molecule in dimeric Kremen1 forms (PDB 5FWV, 5FWS) show as white/grey relative to monomeric form.

In mammals, five sFRPs are known (sFRP1–5). These proteins feature a two-domain structure, containing an N-terminal Fzd-type CRD Frizzled-type cysteine-rich domain (CRD), and a C-terminal netrin-like domain (NLD) [39]. The exact mechanism by which sFRPs function as Wnt signalling inhibitors is still under investigation (in particular, the importance of the NLD in inhibition), but it is widely believed they act as inhibitors by binding Wnt proteins using their CRD, thus preventing the ability of Wnts to bind Fzds and initiate Wnt signalling [40]. Structural knowledge of sFRPs and related proteins is presently limited, with only two such structures reported (Figure 3): the mouse sFRP3 CRD [41] and the *Xenopus* Sizzled protein [42] (Figure 3A). The mouse sFRP3 CRD — along with the mouse Fzd8 CRD — were the first structures of Fzd-type cysteine-rich domains to be characterised. The Sizzled structure is of particular interest as it is the only structure of an sFRP-related protein to feature both the CRD and NLD, providing insight into how the two domains may co-ordinate to modulate Wnt signalling.

### Wnt inhibitory factors (WIFs)

WIFs inhibit Wnt signalling by directly binding the Wnt lipid moiety, to prevent Fzd receptor binding, and prevent Wnt signalling [43]. The structure of the WIF domain of WIF-1 was initially determined by NMR, revealing an immunoglobulin-like fold and the location of the putative lipid-binding site [44] (Figure 3B). The site was subsequently confirmed by X-ray crystallography, as well as the involvement of WIF epidermal growth factor-like domains in binding glycosaminoglycans [45].

### Notum

Notum is an extracellular deacetylase that removes *O*-lipidation from Wnt proteins, thus deactivating them. The structural biology of Notum has primarily been elaborated by a single extensive study [46], wherein structures of human and *Drosophila* Notum bound to *O*-palmitoleylserine, a heparin disaccharide, and the heparin analogue sucrose octasulfate were determined (Figure 3C). *O*-palmitoleylserine is bound by Notum at a hydrophobic cavity deep in the structure. Although a complex with full length Wnt was not determined, the study suggested that the formation of a Wnt-Notum complex is facilitated by heparin binding.

### Dickkopfs (DKKs)

Four mammalian DKKs are known (DKK1-4). These proteins feature two CRDs of a distinct type to that found in sFRPs and Fzds, and primarily act to block canonical Wnt signalling by binding to LRP family co-receptors [47]. DKK also facilitates the Kremen-mediated endocytosis of LRP5/6 [48]. The majority of DKK structures have been determined in complex with LRPs, which will be covered later in the review. Only one structure of an isolated DKK CRD has been determined, that of the second CRD of the mouse DKK2 (Figure 3D) [49].

### Kremens

In conjunction with DKKs, Kremens facilitate blocking of canonical Wnt signalling by promoting the endocytosis of LRPs. The structure of the Kremen1 ectodomain revealed a triangular arrangement of its Kringle, WSC and CUB domains [50] (Figure 3E). The Kringle and WSC domains bind DKK at the opposite face to its LRP-binding interface, while the CUB domain mediates Kremen1 dimerisation in a structure obtained from one of the crystal forms [50]. The WSC domain can also mediate dimerisation (Figure 3E).

## Structure knowledge of Wnt receptors and co-receptors

### Frizzleds (Fzds)

**Figure 4.**
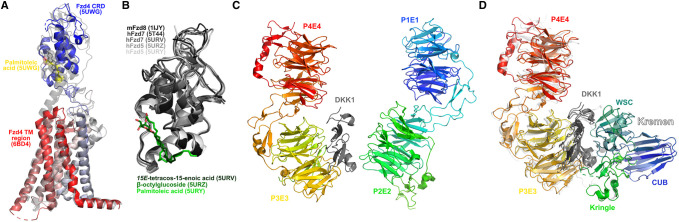
Structures related to transmembrane proteins. (**A**) Frizzled-4 model generated by overlay of Frizzled-4 cysteine-rich domain (CRD) bound to palmitoleic acid (PDB 5UWG) and Frizzled-4 transmembrane (TM) region (PDB 6BD4) to Smoothened bound to cholesterol (PDB 5L7D; transparent grey). (**B**) Representative Frizzled CRD structures and complexes. Structures depicted include: native mouse Fzd8 CRD (PDB 1IJY), native human Fzd7 (PDB 5T44), human Fzd7 bound to *15E*-tetracos-15-enoic acid (PDB 5URV), human Fzd5 bound to β-octylglucoside (PDB 5URZ), human Fzd5 bound to palmitoleic acid (PDB 5URY). (**C**) Atomic structure of LRP6 ectodomain constructed from fitting X-ray structures of P1E1 and P2E2 regions (PDB 4DG6) and the complex of DKK1 with P3E3 and P4E4 regions (PDB 3S2K) to the electron microscopy structure (PDB 5GJE). Legend: red-to-blue rainbow — LRP6 N-to-C-terminal; grey — DKK1. (**D**) LRP6–DKK-Kremen interactions. LRP6–DKK1–Kremen1 complex (PDB 5FWW) depicted in colours; LRP6–DKK1 complex (PDB 3S2K), unbound DKK1 (PDB 2JTK) and unbound Kremen1 (PDB 5FWT) overlaid to PDB 5FWW and depicted in transparent grey.

Together with the related Smoothened receptor, which mediates Hedgehog signalling, the Frizzleds form a class of G protein-coupled receptors that feature a seven-helical transmembrane domain (as per other GPCRs) and a distinctive cysteine-rich ectodomain (CRD) used to bind ligands. The structures of the CRDs of Fzd2 [51], Fzd4 [30,31,52,53], Fzd5 [54,55], Fzd7 [52,54–56] and Fzd8 [41,52,57] are presently represented in the Protein Data Bank; only a single Fzd transmembrane domain structure, that of Fzd4 [58], is presently known (Figure 4A,B). No structures featuring both the CRD and TM regions of Fzds are presently available. However, the structural biology of the Fzds can be inferred from that of the more extensively structurally characterised Smoothened [59–66]. Of particular note is the recent determination of Smoothened in an active conformation bound to a G protein, revealing a similar opening of the intracellular regions of the receptor to that observed in other classes of GPCRs; such opening was earlier inferred to occurred in Fzds [67].

### Low-density lipoprotein receptor-related proteins 5 and 6 (LRP5/6)

LRP5 and LRP6 function as Wnt ligand co-receptors in canonical Wnt signalling, and are antagonised by DKKs and Kremens. The ectodomain of LRP6 has been extensively structurally characterised and is primarily defined by the presence of four 7-bladed β-propellers followed by EGF-like domains (P1E1, P2E2, P3E3, P4E4) (Figure 4C). The structure of the combination of the LRP6 P1E1 and P2E2 regions [68], as well as that of the P3E3 and P4E4 regions [68–70], have been determined by X-ray crystallography, while the structure of the complete ectodomain has been determined by electron microscopy [69,71]. DKK1 has been structurally demonstrated to bind at the P1E1 [72] and P3E3 [50,68,70] regions, with Kremen binding on the opposite face of DKK to LRP, allowing formation of a ternary complex (Figure 4D).

## Structural knowledge of intracellular proteins and complexes mediating Wnt signalling

### Dishevelleds (Dvls)

Dvls feature three ordered domains: an N-terminal DIX domain, promoting Dvl oligomerisation into signalosomes, as well as mediating Axin binding; a PDZ domain, facilitating interactions with various proteins as well as weakly contributing to Fzd binding (although demonstrated to be dispensable for canonical Wnt signalling [73]); and a DEP domain, facilitating high affinity interaction with Fzds [74], as well as Fzd endocytosis [75]. These domains of Dvl1 and Dvl2 have been the focus of all currently published structures.

**Figure 5.**
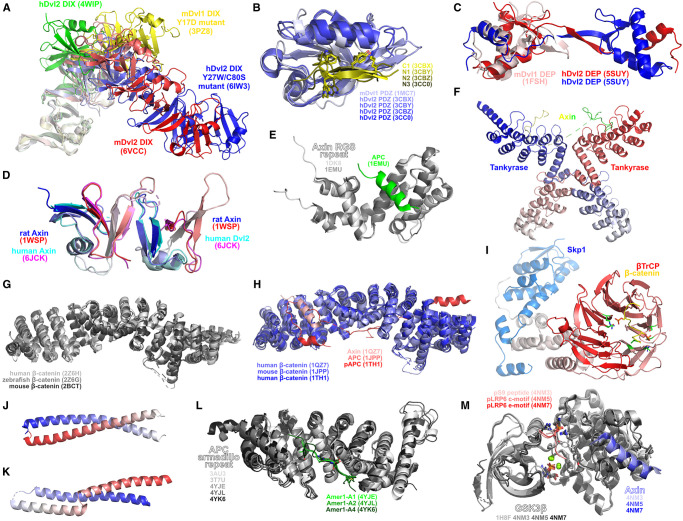
Structures related to cytoplasmic proteins and interactions. (**A**) Dvl DIX domain oligomeric structures. (**B**) Dvl PDZ domain. Structures represented include: native mouse Dvl1 PDZ (PDB 1MC7), human Dvl2 PDZ in complex with C1 inhibitory peptide (PDB 3CBX), human Dvl2 PDZ in complex with N1 inhibitory peptide (PDB 3CBY), human Dvl2 PDZ in complex with N2 inhibitory peptide (PDB 3CBZ), human Dvl2 PDZ in complex with N3 inhibitory peptide (PDB 3CC0). The structure of the N2 complex with Dvl2 PDZ was inferred via the generation of a symmetry-related dimer. Selected residues with similar chemical functionality across multiple peptides — loosely highlighting how Fzd KTxxxW motifs may be recognised by Dvl PDZ domains — are shown as sticks. (**C**) Dvl DEP domain. Structures represented include: native mouse Dvl1 DEP domain (PDB 1FSH); human Dvl2 DEP domain dimer crystallised from dimeric fraction (PDB 5SUY). (**D**) Axin DIX domain. Structures represented include: rat Axin homodimer (PDB 1WSP); human Axin-Dvl2 heterodimer (PDB 6JCK). Each molecule coloured from N-to-C-terminal in blue-to-red/cyan-to-magenta gradient. (**E**) Axin RGS repeat in unbound (PDB 1DKS) and APC-bound (PDB 1EMU) states. (**F**) Mouse tankyrase-axin complex (PDB 3UTM). Each tankyrase monomer is coloured from N-to-C terminal as blue/red to white gradient. Dashes indicate missing portions of the axin structure. (**G**) Native β-catenin structures. (**H**) Complexes of interactions of cytoplasmic β-catenin. Structures represented include: human β-catenin bound to *Xenopus* Axin (PDB 1QZ7); mouse β-catenin bound to an APC fragment (PDB 1JPP); human β-catenin bound to a phosphorylated human APC fragment (PDB 1TH1). (**I**) APC N-terminal coiled-coil region (residues 2–55) (PDB 1DEB). Each chain coloured from N-to-C terminal as blue/red to white gradient. (**J**) APC N-terminal helical region (residues 126–250) (PDB 1M51). Coloured from N-to-C terminal as blue-white-red gradient. (**K**) APC armadillo repeat. Structures represented include: native APC (PDB 3AU3 and 3T7U); APC in complex with Amer1-A1 (PDB 4YJE); APC in complex with Amer1-A2 (PDB 4YJL); APC in complex with Amer1-A4 (PDB 4YK6). (**L**) β-TrCP-Skp1-β-catenin complex (PDB 1P22). Phosphate-contacting residues in β-TrCP are shown green. Phosphorylated β-catenin residues and their contacts in β-TrCP shown as sticks. (**M**) GSK3β complexes elaborating Wnt signalling. Structures represented include: *apo*-GSK3β (PDB 1H8F); GSK3β bound to N-terminal autoinhibitory phosphopeptide (pS9) and Axin (PDB 4NM3); GSK3β bound to phosphorylated LRP6 c-motif and Axin (PDB 4NM5); GSK3β bound to phosphorylated LRP6 e-motif and Axin (PDB 4NM7). Bound ADP, phosphorylated residues on peptides and Arg96, Arg180, Lys205 and Tyr216 shown as sticks. Magnesium shown as green spheres.

Structural characterisation of wildtype and mutant DIX domains of human Dvl2, mouse Dvl1 and mouse Dvl2 reveal in all instances the formation of a superhelical oligomeric structure [76–79]; the pitch of the superhelix varies appears to vary according to the specific DIX domain being examined, as well as through the introduction of interface mutants (Figure 5A). The variety of Dvl PDZ structures determined illustrate the flexibility of the domain's binding pocket to accommodate various ligands (Figure 5B) [80–83]. In particular, a series of structures of the human Dvl2 PDZ in complex with several peptides derived from phage display suggests how Dvl PDZ domains may recognise the C-terminal KTxxxW motifs contained in Fzds [81]. The structure of the mouse Dvl1 DEP domain was the first of any Dvl domain to be solved, demonstrating a fold exhibiting a strong electric dipole suggested to facilitate membrane targeting [84]. While the Dvl DEP domain has been illustrated to afford a key role in directly binding Fzds [74], the most recent structural evidence for any Dvl DEP domain — that of the human Dvl2 in a domain-swapped dimeric configuration — illustrate a potential role for the DEP domain in assembling Wnt signalosomes, as well as in mediating signal directionality [85] (Figure 5C).

### Axin and tankyrase

Axin, like Dvl, contains a DIX domain which can undergo head-to-tail oligomerisation (Figure 5D). Recently, a complex between the Axin DIX domain and the Dvl2 DIX domain has been determined [86], revealing a similar structure of the Axin-Dvl heterodimer compared with both the Axin DIX homodimer [87] and Dvl homomer structures [76–79]. Although extended heterooligomer structures have not been demonstrated, these are presumed to form a superhelical structure with a varied pitch compared to the currently determined Dvl DIX homooligomer structures. Axin also directly interacts and has been structurally characterised with APC [88], GSK3β [89], β-catenin [90] and tankyrase. With the exception of its interaction with APC (Figure 5E), Axin utilises short segments to interact with these proteins (Figure 5F,H,L). The complex of Axin with the tankyrase ankyrin repeat reveals that the N-terminal of Axin binds to tankyrase in a 1:2 fashion (Figure 5F) [91].

### β-catenin

The structures of the armadillo repeat regions of human [92], mouse [93] and zebrafish [92] β-catenin have been characterised, revealing a relatively conserved and remarkably rigid structure (Figure 5G). Complexes of this region of β-catenin with APC [90,94,95] and axin [88,96,97] have also been determined (Figure 5H). Axin utilises a short helical fragment to bind to armadillo repeats 3 and 4 of β-catenin, while APC uses an extended region to interact with approximately the entire length of β-catenin. Although part of APC binds to β-catenin at an overlapping region to Axin, APC does not share Axin's helical secondary structure in this location, indicating β-catenin's ability to bind peptides distinct in sequence and structure. N-terminal phosphorylation of β-catenin facilitates its destruction, and complexes of the phosphorylated N-terminal of β-catenin with the SCF ubiquitin ligase β-TrCP-Skp1 have been determined. These indicate that the phosphorylated N-terminal of β-catenin interacts with β-TrCP at the opposite face of the β-propeller to Skp1, with pSer33 bound by the first and second blades of the β-propeller and pSer37 bound by the fifth blade (Figure 5I) [98,99].

### Adenomatous polyposis coli protein (APC)

APC is a very large protein comprising, in simplest terms, an N-terminal leucine-rich region (residues 1–730) and a C-terminal serine-rich region (residues 731–2832). Short fragments from the C-terminal serine-rich region have been structurally demonstrated in complex with a range of proteins, including axin (Figure 5E), β-catenin (Figure 5H), the Src-homology 3 domain of DDEF1 [100], and the PDZ1 [101] and PDZ2 [102] domains of DLG1. The N-terminal leucine-rich region contains at least three helical regions that have been structurally characterised: an N-terminal dimeric coiled-coil domain (residues 2–55) [103] (Figure 5J), an helical region forming a monomeric coiled-coil (residues 126–250) [104] (Figure 5K) and a series of armadillo repeats (residues 453–767) [105–109]. The N-terminal dimeric coiled-coil is poorly stable in isolation, suggesting that the dimerisation motif may extend beyond the first 55 amino acids of APC, although it is unclear whether the monomeric coiled-coil that follows contributes to dimerisation. The APC armadillo repeat region has been structurally characterised with several fragments of Amer1 (Figure 5L). These structures reveal that Amer1 fragments use a relatively functionally conserved motif to bind APC, consisting of Ser/Thr/Tyr to bind armadillo repeats 4–6 and a small polar amino acid (Gly/Ser/Cys) followed immediately by a glycine and a negatively charged amino acid to bind repeats 2–4. Hydrophobic amino acids (typically a longer chain aliphatic amino acid followed by alanine) bind repeats 1–3.

### Glycogen synthase kinase 3β (GSK3β)

While GSK3β has been extensively structurally characterised as part of many medicinal chemistry research programs, a small selection of structures provide specific insight into its role in modulating Wnt signalling (Figure 5M). X-ray crystal structures of GSK3β in complex with the minimal binding segment of Axin [89] illustrate that Axin utilises an α-helical segment to bind GSK3β. The structures of GSK3β bound to its phosphorylated autoinhibitory peptide and phosphorylated LRP6 motifs illustrate the importance of conformational changes in regulating GSK3β function and how primed substrates are recognised by GSK3β [15]. Specifically, the loop from residues 89-95 moves from the open conformation observed in the unbound state [110] to clamp onto the peptide, the phosphorylated residue is bound by three positively charged residues — Arg96, Arg180 and Lys205 — and Tyr216 rotates to facilitate peptide access to the active site.

## Structural knowledge of intranuclear proteins and complexes mediating Wnt signalling

### Nuclear β-catenin

**Figure 6.**
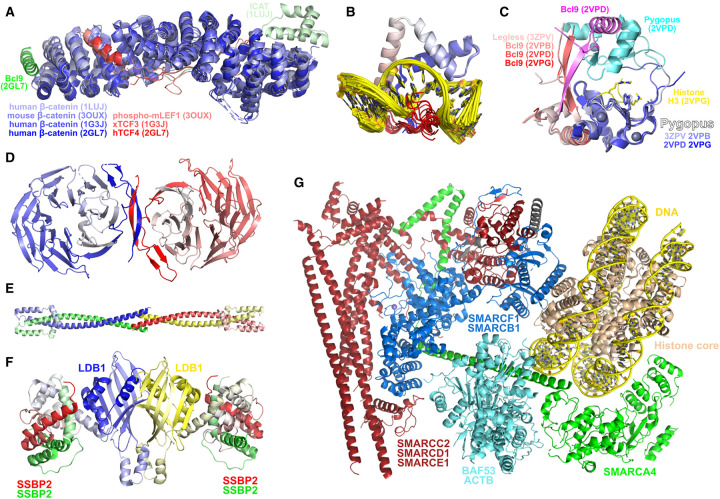
Structures related to intranuclear proteins and interactions. (**A**) Nuclear β-catenin interactions. Structures represented include: human β-catenin bound to mouse ICAT (PDB 1LUJ), mouse β-catenin bound to phosphorylated mouse LEF1 (PDB 3OUX), human β-catenin bound to *Xenopus* TCF3 (PDB 1G3J), ternary complex of human β-catenin, human TCF4 and human Bcl9 (PDB 2GL7). (**B**) Mouse LEF1 high mobility group domain (blue–red N-to-C-terminal) bound to DNA (yellow) (PDB 2LEF). (**C**) Pygopus–Bcl9 interactions. Structure represented include: *Drosophila* Pygopus–Legless complex (PDB 3ZPV), human Pygopus1–Bcl9 complex (PDB 2VPB), human Pygopus1–Bcl9 dimer of dimers (PDB 2VPD), human Pygopus1–Bcl9-histone H3 tail ternary complex (PDB 2VPG). (**D**) TLE1 WD repeat dimer (PDB 1GXR). Legend: blue-white — molecule 1 N-to-C terminal; red-white — molecule 2 N-to-C terminal. (**E**) TLE1 Q domain tetramer (PDB 4OM2). Each chain coloured from N-to-C terminal in blue/red/yellow/green to white. (**F**) Assembly of the ChiLS complex in 4:2 stoichiometry based on available structures (PDB 6TYD and PDB 6S9S). (**G**) Structure of nucleosome-bound human BAF complex (PDB 6LTJ).

Structures of TCFs [111–114] and LEF-1 [115] in complex with β-catenin (Figure 6A) reveal that β-catenin wraps around the N-terminal of these proteins, utilising approximately the full length of the armadillo repeats to bind the transcription factors, similar to how APC is bound by β-catenin (Figure 5H). Bcl9 binds to the first armadillo repeat of β-catenin using a short helix located between proline-rich stretches of its sequence, while the β-catenin inhibitor ICAT uses a small N-terminal helical domain to bind the final armadillo repeats of β-catenin, and a C-terminal extension that overlaps with much of the TCF/LEF binding site, thus blocking TCF/LEF binding [97,116]. TCFs and LEFs utilise a high mobility group (HMG) box domain to bind DNA; the structure of the mouse LEF-1 HMG box domain bound to DNA was one of the earliest structures of such a domain, as well as a DNA–HMG box complex, and illustrates the bending of the DNA double helix characteristic of DNA–HMG box interactions (Figure 6B) [117].

### B-cell CLL/lymphoma 9 protein (Bcl9) and Pygopus

Bcl9 forms a ternary complex with β-catenin and TCF transcription factors, binding at a distinct site on β-catenin to TCF, as well as other β-catenin-interacting proteins [111,118]. The function of Bcl9 is enhanced via binding to Pygopus proteins and their homologues, wherein a helical segment of Bcl9 interacts with the PHD-type zinc finger of Pygopus [119,120]; the Bcl9-like protein (BCL9L) forms a similar complex with Pygopus [121,122]. The human Bcl9–Pygopus heterodimer has been characterised in complex with a methylated histone fragment, illustrating the importance of Trp366 in Pygopus in interacting with methylated arginine and lysine; this residue is substituted for a phenylalanine in *Drosophila* Pygopus and likely facilitates similar interactions. Additionally, Bcl9 and Pygopus have been demonstrated to form a dimer of heterodimers; such an arrangement appears compatible with the binding of methylated histones (Figure 6C).

### Groucho family proteins

Structures of the human Groucho family protein TLE1 have been obtained for its C-terminal WD repeat region, a seven-bladed β-propeller forming a dimer mediated by its N-terminal segment [123,124] (Figure 6D). The N-terminal Q domain, which mediates TCF binding, forms a dimeric coiled coil which in turn dimerises in a head-to-head fashion to give the active tetrameric species [125] (Figure 6E).

### ChiLS complex

The biological assembly of the LUFS domain of the human SSDP2 revealed a tightly packed tetramer formed by dimerisation of dimers [126]. The biological assembly of the *Xenopus* LDB1 bound to darpin 10 illustrates the dimerisation of LDB proteins [21]. The biological assembly of the human SSDP2 in complex with the human LDB1 illustrates a 2:1 stoichiometry between SSDP2 and LDB1, with LDB1 binding at the tetramerization interface of SSDP2 [127]; this in turn suggests that the SSDP2 tetramer previously determined may represent an inactive state. Judicious overlay of the presently determined structures allows the development of a structural model of the ChiLS complex, displaying the determined 4 : 2 stoichiometry between the SSDP and LDB components [21] (Figure 6F).

### BAF complex

The BAF complex is a very large complex comprised of numerous subunits that functions as a Wnt transcriptional co-activator. The SWI/SNF-related matrix-associated actin-dependent regulator of chromatin (SMARC) subfamily members, which are key components of this complex, have been the subject of numerous structural biology [128–130] and medicinal chemistry [131–133] efforts. Very recently, the structure of a nucleosome-bound human BAF complex has been determined [134] (Figure 6G). This structure reveals that SMARCC2 forms a dimeric coiled-coil, with which helical regions of SMARCD1 and SMARCE1 interact and which likely forms a scaffold for the complex. SMARCF1 contains an armadillo repeat-like region that interacts with this helical scaffold on one face and with the N-terminal domain of SMARCB1 with its opposing face. The SMARCB1 C-terminal domains interact with the SWIRM domains of both SMARCC1 molecules, an interaction that appears further stabilised by BAF45D. This assembly positions SMARCB1 to interact directly with histones H2A and H2B on one face of the nucleosome. SMARCA4 adopts a highly extended conformation, interacting with almost all subunits of the complex, cradling the opposite face of the nucleosome to SMARCB1 with its helicase domains. The extended conformation and nucleosome-binding by SMARCA4 appear to be supported through interaction with actin-like protein 6A (BAF53) and cytoplasmic actin 1 (ACTB).

## Future challenges in the structural biology of Wnt signalling

Wnt structural biology has considerably grown in the past 20 years, however, there are still a number of notable gaps in knowledge. These include the structure of an active Wnt signalosome and/or components thereof (e.g. full length Fzd, Fzd in an active conformation, Fzd bound to Dvl), an overall view of the Wnt degradosome, and a comprehensive understanding of the structure of the Wnt enhanceosome. Cryoelectron microscopy, which has facilitated the structural determination of many targets that were typically challenging or thought impossible by X-ray crystallography (including very large protein complexes and membrane-bound proteins in various states), has the potential to fill these gaps in structural knowledge of Wnt signalling. Nonetheless, significant protein engineering is likely to be required to achieve constructs sufficiently stable for structure determination, as has facilitated the elaboration of membrane protein structure and pharmacology. Computational approaches may also be valuable to fill some of these gaps — in particular, the combinatorial range of potential protein–protein interactions regulating the earlier stages of the pathway. The present structural data on canonical Wnt signalling affords numerous opportunities for structure-based drug design, with the recent growth allowing further dissection and effective targeting of this fascinating pathway.

## Perspectives

- Canonical/β-catenin-dependent Wnt signalling is a pathway of enormous interest as a potential target in cancer treatment, as well as being crucial in the early stages of development.
- Structural knowledge of proteins and interactions involved in facilitating and antagonising canonical Wnt signalling has grown considerably over the past 20 years.
- Major frontiers to conquer relate primarily to understanding the assembly of large multiprotein complexes mediating Wnt signalling — in particular, the structure, activation and interactions of membrane receptors, as well as the assembly of nuclear proteins.

## Open Access

Open access for this article was enabled by the participation of University of Edinburgh in an all-inclusive *Read & Publish* pilot with Portland Press and the Biochemical Society under a transformative agreement with JISC.

## Abbreviations

APC: Adenomatous Polyposis Coli
BCL9: B-cell CLL/lymphoma 9 protein
ChiLS: Chip-SSDP/LIM-domain binding protein
CK1α: Casein kinase 1α
CRD: Cysteine-rich domain
CUB: Complement C1r/C1s, Uegf, Bmp1
Dkk: Dickkopf proteins
Dvl: Dishevelled
Fzd: Frizzled receptor
GSK3β: Glycogen synthase kinase 3β
HMG: High mobility group
LGR: Leucine-rich repeat-containing G-protein coupled receptor
LRP: low-density lipoprotein receptor
LRR: leucine-rich repeat
NLD: Netrin-like domain
PP2A: Protein phosphatase 2A
RSPO: R-spondin
SCF: Skp-Cullin-F-box
sFRP: secreted-Frizzled related protein
SMARC: SWI/SNF-related matrix-associated actin-dependent regulator of chromatin
TCF/LEF: T-cell factor/lymphoid enhancer factor
WIF: Wnt inhibitory factor
WSC: Cell wall integrity and stress response components
